# Plasmon-mediated resonance energy transfer by metallic nanorods

**DOI:** 10.1186/1556-276X-8-209

**Published:** 2013-05-03

**Authors:** Yi-Cong Yu, Jia-Ming Liu, Chong-Jun Jin, Xue-Hua Wang

**Affiliations:** 1 State Key Laboratory of Optoelectronic Materials and Technologies, School of Physics and Engineering, Sun Yat-sen University, Guangzhou 510275, China

**Keywords:** Resonance energy transfer, Silver nanorods, Surface plasmons

## Abstract

**Abstract:**

We investigate the enhancement of the resonance energy transfer rate between donor and acceptor associated by the surface plasmons of the Ag nanorods on a SiO_2_ substrate. Our results for a single nanorod with different cross sections reveal that the cylinder nanorod has the strongest ability to enhance the resonance energy transfer rate. Moreover, for donor and acceptor with nonparallel polarization directions, we propose simple V-shaped nanorod structures which lead to the remarkable resonance energy transfer enhancement that is ten times larger than that by the single nanorod structure. We demonstrate that these structures have good robustness and controllability. Our work provides a way to improve the resonance energy transfer efficiency in integrated photonic devices.

**PACS:**

78.67.Qa, 73.20.Mf, 42.50.Ex

## Background

Resonance energy transfer (RET) between nanosystems is extensively researched in nanophotonics, which has various important applications ranging from biological detections and chemical sensors to quantum information science [1-11]. RET may proceed in different transfer distances: the Dexter process [12] based on wave function overlap transfers within the range of about 1 nm, and the Forster process [13] caused by the near-field resonant dipole-dipole interaction transfers usually within the range of 10 nm. The efficient transfer energy distance is still very short. It is thus important to enhance the efficiency of RET in a long distance.

The RET rate by the dipole-dipole interactions can be greatly manipulated by the electromagnetic environment; many different kinds of electromagnetic environments have been used to enhance the resonant dipole-dipole interaction strength and the efficiency of the RET, such as optical cavities [2,14-17], optical lens or fiber [18,19], and metamaterials [20,21]. In the last decades, it has been demonstrated that surface plasmon supported by metal nanostructures is a powerful tool to enhance the efficiency of RET. Since Andrew et al. [5] demonstrated long-distance plasmon-mediated RET using Ag films, a great deal of efforts have been devoted to investigate plasmon-mediated RET using nanoparticles [22-25], plasmonic waveguides [9,11,26], single nanowires [27-30], and nanorod or nanowire arrays [10,19,31]. Most of the previous works focus on the case of the donor and acceptor having parallel transition dipole moments. However, in practical devices, it is extremely difficult to satisfy the parallel condition between the dipole moments of the donor and acceptor, and when the donor and acceptor have nonparallel dipole moments, the RET rate may decrease evidently. It is thus important to design nanostructures to achieve big RET enhancement for donor and acceptor with nonparallel dipole moments.

In this paper, we investigate the enhancement of the RET rate between donor and acceptor associated by surface plasmons of Ag nanorods on a SiO_2_ substrate. Firstly, we consider single nanorods with different cross sections, and the results reveal that the cylinder nanorod has the strongest ability to enhance the RET rate. We also find that the enhancement of RET rate in the single nanorod structure decreases when the donor and acceptor have nonparallel dipole moment directions. We then propose simple V-shaped nanorod structures for a donor-acceptor pair with nonparallel dipole moments. We find that these structures can lead to a remarkable resonance energy transfer enhancement ten times larger than that by the single nanorod structure. We demonstrate that the enhancing effect by these structures can be controlled by the nanorod length of the branch in the V-shaped structure and that these structures are robust regardless of the shape and material of the corner part. This controllability and robustness are also preserved for donor-dipole pair with asymmetric configuration. Therefore, these structures can be applied in integrated photonic devices.

## Methods

Without the loss of generality, we quantify the enhancement of RET by the normalized energy transfer rate (nETR), which means that the RET rate normalized to the case in vacuum. The nETR is given as [32,33]

(1) $$\begin{array}{l} \mathrm{nETR}=\frac{{\left|n_{A}\cdot G\left(r_{A},\;r_{D},\omega \right)\cdot n_{D}\right|}^{2}}{{\left|n_{A}\cdot G_{\mathrm{vac}}\left(r_{A},\;r_{D},\omega \right)\cdot n_{D}\right|}^{2}}\\ \;=\frac{{\left|n_{A}\cdot E_{D}\left(r_{A},\;\omega \right)\right|}^{2}}{{\left|n_{A}\cdot E_{D,\mathrm{vac}}\left(r_{A},\;\omega \right)\right|}^{2}}, \end{array}$$

where **n**_*A*_ and **n**_*D*_ are the unit vectors along the directions of the dipole moments of the acceptor and donor, respectively, *ω* is the transition frequency, **G**(**r**_*A*_, **r**_*D*_, *ω*) is the dyadic Green's function [34], **E**_*D*_(**r**_*A*_, *ω*) is the electric field at the position of the acceptor induced by the donor dipole in the presence of the plasmonic structures, while **G**_vac_(**r**_*A*_, **r**_*D*_, *ω*) and **E**_*D*,vac_(**r**_*A*_, *ω*) correspond to the case in vacuum but without the plasmonic structures.

The calculations of the electric field induced by the dipole are performed by the finite element method with the commercial COMSOL Multiphysics software. All metal structures in this paper are set to be silver; the electric permittivity of silver is gathered by fitting the experimental data of Johnson and Christy with piecewise cubic interpolation [35]. All nanostructures are set on a semi-infinite SiO_2_ substrate with the refractive index of 1.456, and the surrounding medium is air.

## Results and discussion

**Figure 1:**
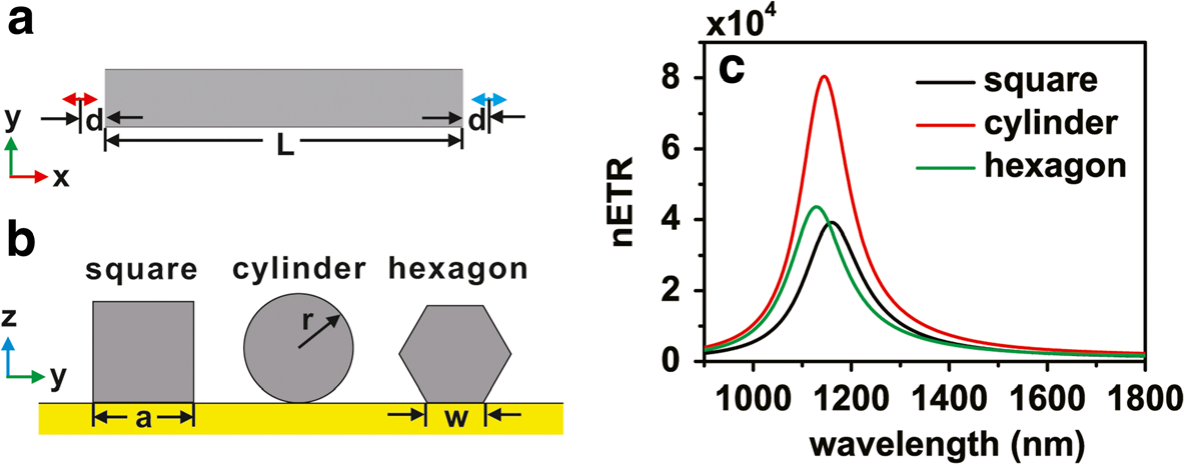
**Structure diagram and nETR for single nanorods with different cross sections.** (**a**) Schematic picture on an *xy* plane. (**b**) Cross sections of the different nanorods on a *yz* plane. (**c**) The nETR for square nanorod with *a* = 40 nm (black), cylinder nanorod with *r* = 20 nm (red), and hexagon nanorod with *w* = 25 nm (green). The distance between both dipoles and the ends of the nanorods is *d* = 20 nm, and the longitudinal length of the nanorods is *L* = 250 nm.

Firstly, we consider single Ag nanorod structures with different cross sections. The schematic pictures of the single nanorod structures and their cross sections are shown in Figure 1a,b. The donor and acceptor dipoles are both aligned to the center axis of the nanorod at different ends, the distance from each dipole to the end of the nanorod is *d* = 20 nm, and the longitudinal length of the nanorods is set to *L* = 250 nm. Notice that the longitudinal surface plasmon resonance modes of the nanorods are responsible for the enhancement of the RET rate; in order to compare the ability of different nanorods to enhance the RET, we tune the parameters *a*, *r*, and *w* to make the resonance frequencies of their longitudinal surface plasmon modes approximately equal. Figure 1c displays the nETR spectra for these three structures, with *a* = 40 nm for the square nanorod, *r* = 20 nm for the cylinder nanorod, and *w* = 25 nm for the hexagon nanorod. It can be seen that in the wavelength range between 1,050 nm and 1,275 nm, all three structures support the enhancement of RET over 10^4^. The nETR spectrum for the square nanorod has a peak at about 1,160 nm with an enhancement of about 39,200. For the hexagon nanorod, the nETR spectrum has a peak at 1,130 nm with an enhancement of 43,600. Moreover, in the whole wavelength range from 900 to 1800 nm, the nETR in the cylinder nanorod structure is always greater than those in the other two structures; it has a peak at 1,145 nm with an enhancement of nearly 80,400. This indicates that the cylinder nanorod has the strongest ability to enhance the RET rate by its longitudinal surface plasmon resonances. We note that among these three structures, the cylinder nanorod has the highest symmetry; this may improve the coupling between the dipoles and the surface plasmons and then increase the RET rate. Although the cylinder nanorod can lead to a nETR that is twice than that in the square nanorod, the fabrication of the cylinder nanorod on the substrate is much more difficult. The square nanorod should still be the primary choice in practical applications.

**Figure 2:**
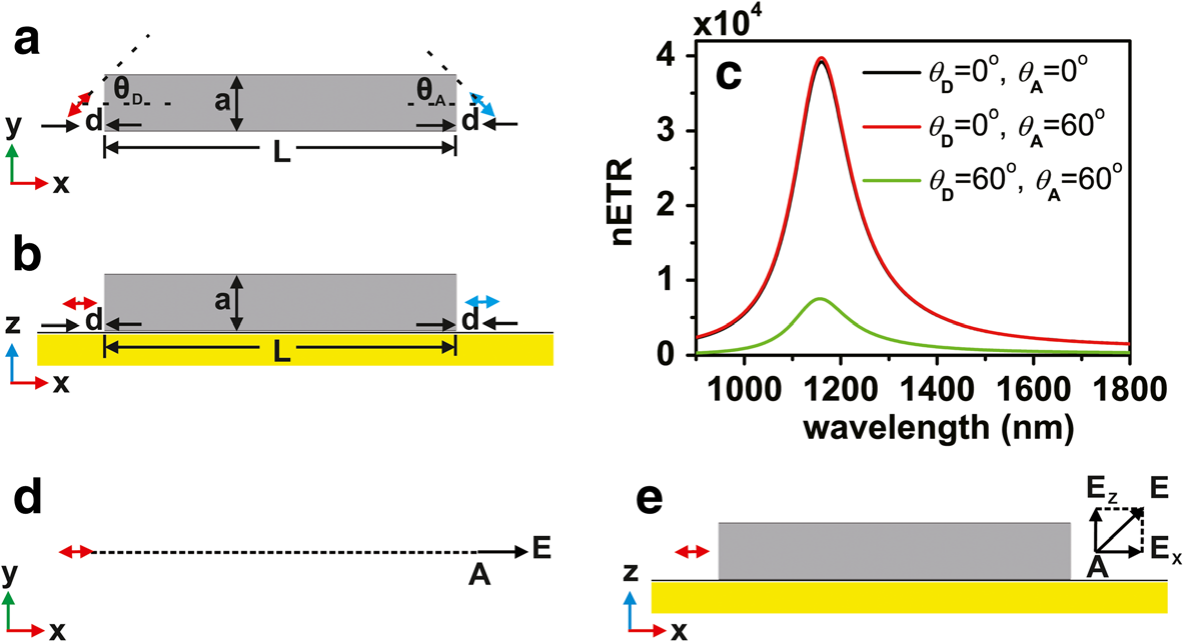
**Energy transfer between donor and acceptor with different dipole moment directions in single square nanorod.** (**a**) Schematic picture on *xy* plane. (**b**) Schematic picture on *xz* plane. (**c**) The nETR with *a* = 40 nm, *d* = 20 nm, *L* = 250 nm, and different values of *θ*_*D*_ and *θ*_*A*_. The schematic pictures for the electric field at the position of the acceptor induced by the donor with *θ*_*D*_ = 0° (**d**) in vacuum and (**e**) in the nanorod structure.

We now turn to investigate the nETR for donor and acceptor having nonparallel dipole moments. Figure 2a,b displays the schematic pictures of the structure. Here we choose the square nanorod. The angle between the dipole moment of the donor and the principle axis of the nanorod is denoted as *θ*_*D*_, while the angle between the dipole moment of the acceptor and the principle axis of the nanorod is denoted as *θ*_*A*_. The nETR spectra for different *θ*_*D*_ and *θ*_*A*_ are displayed in Figure 2c, with *a* = 40 nm, *L* = 250 nm, and *d* = 20 nm. It can be seen that the red curve corresponding to the nonparallel case of *θ*_*D*_ = 0° and *θ*_*A*_ = 60° is overlapped with the black curve of the parallel case of *θ*_*D*_ = 0° and *θ*_*A*_ = 0°. To comprehend it, we notice that **n**_*A*_ only has *x*-direction and *y*-direction components. According to Equation 1, the nETR is determined by the angle *θ*_*A*_ together with the *x*-direction and *y*-direction components of the electric field at the position of the acceptor induced by the donor dipole. When we keep *θ*_*D*_ = 0°, the donor dipole is directly pointing at the acceptor. When the dipoles are in vacuum, as shown in Figure 2d, the electric field **E**_*D*,vac_(**r**_*A*_) is along the *x*-direction, and its *y*-direction and *z*-direction components vanish. When the dipoles are in the nanorod structure, the donor dipole direction is along the principle axis of the nanorod and is thus in the symmetry plane of the structure. According to the symmetry, the *y*-direction component of electric field **E**_*D*_(**r**_*A*_) also vanishes, as shown in Figure 2e. Therefore, only the *x*-direction components of the electric fields contribute to the RET rates; for different *θ*_*A*_ values, we have

(2) $$\begin{array}{l} \frac{{\left|n_{A}\left(60^{\circ }\right)\cdot E_{D}\left(r_{A},\;\omega \right)\right|}^{2}}{{\left|n_{A}\left(0^{\circ }\right)\cdot E_{D}\left(r_{A},\;\omega \right)\right|}^{2}}=\frac{{\left|n_{A}\left(60^{\circ }\right)\cdot E_{D,\mathrm{vac}}\left(r_{A},\;\omega \right)\right|}^{2}}{{\left|n_{A}\left(0^{\circ }\right)\cdot E_{D,\mathrm{vac}}\left(r_{A},\;\omega \right)\right|}^{2}}\\ \;=\cos^{2}60^{\circ }=\frac{1}{4}. \end{array}$$

It is thus straightforward to get

(3) $$\begin{array}{l} \frac{{\left|n_{A}\left(60^{\circ }\right)\cdot E_{D}\left(r_{A},\;\omega \right)\right|}^{2}}{{\left|n_{A}\left(60^{\circ }\right)\cdot E_{D,\mathrm{vac}}\left(r_{A},\;\omega \right)\right|}^{2}}\\ \;=\frac{{\left|n_{A}\left(0^{\circ }\right)\cdot E_{D}\left(r_{A},\;\omega \right)\right|}^{2}}{{\left|n_{A}\left(0^{\circ }\right)\cdot E_{D,\mathrm{vac}}\left(r_{A},\;\omega \right)\right|}^{2}}, \end{array}$$

resulting in the same nETR shown in Figure 2c. While for the case of *θ*_*D*_ = 60° and *θ*_*A*_ = 60°, it can be seen that the nETR decreases evidently, the resonance wavelength is about 1,157 nm, and the maximum enhancement is reduced to about 7,500. The above results demonstrate that, in order to produce high RET enhancement in the single nanorod structure, the direction of the donor or acceptor dipole should be along the principle axis of the nanorod, otherwise the enhancement decreases evidently.

**Figure 3:**
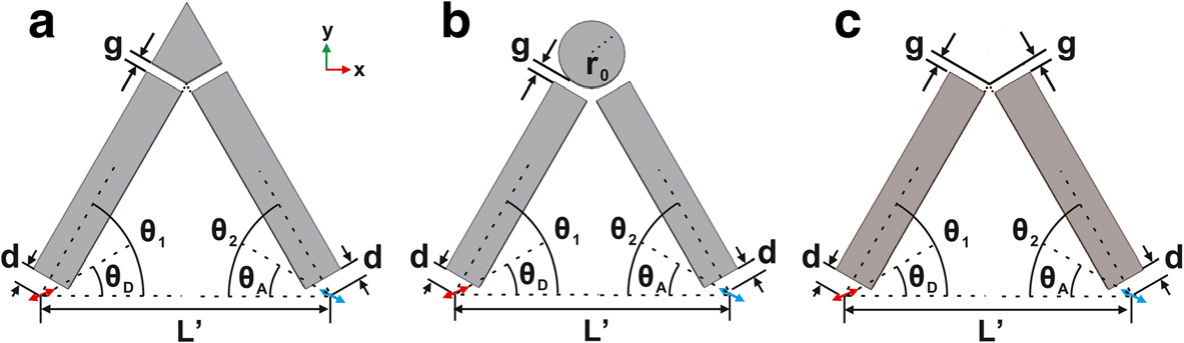
**Schematic cross-sectional pictures of the V-shaped nanorod structures.** With a (**a**) sharp corner part, (**b**) cylinder corner part, and (**c**) no corner part, respectively.

**Figure 4:**
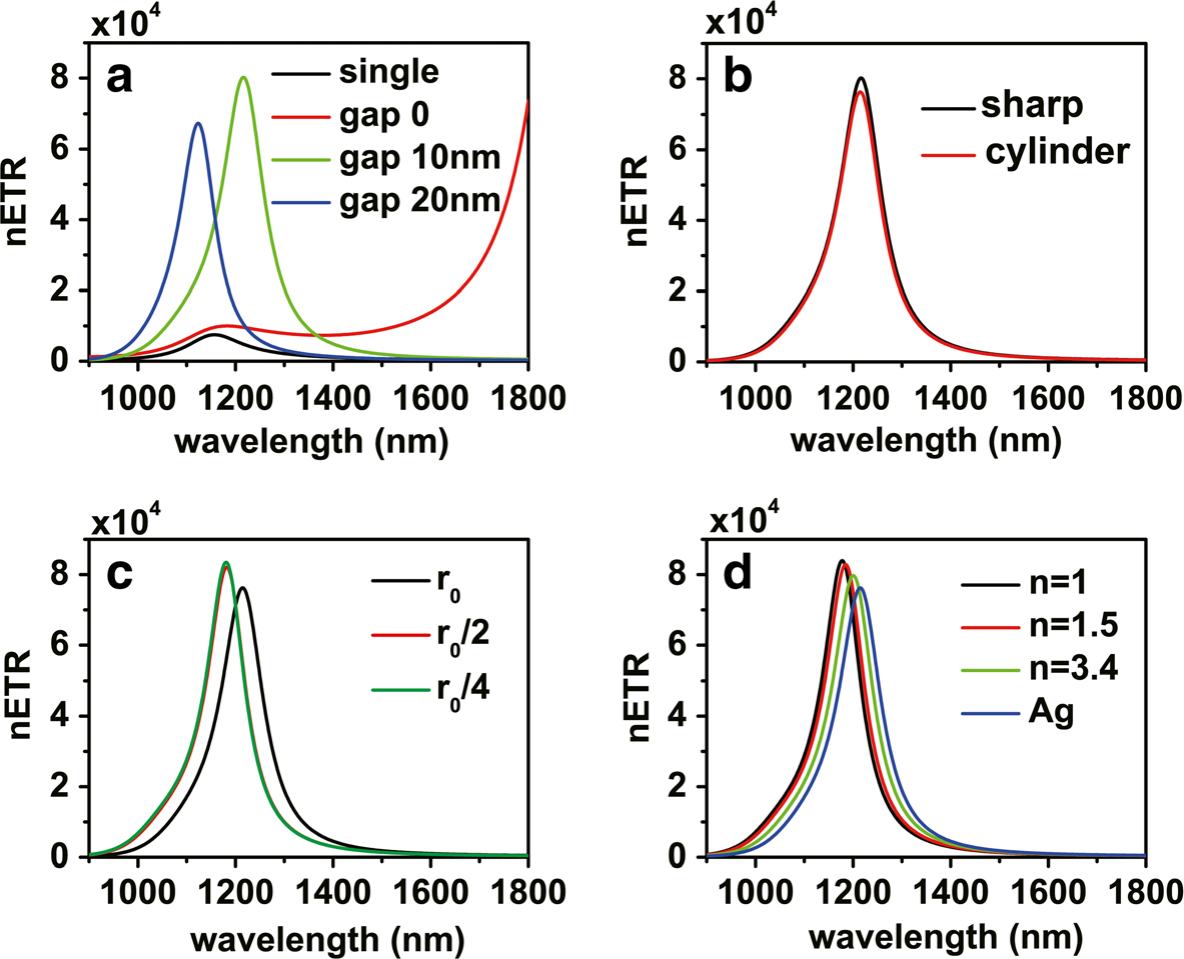
**The nETR spectra for different V-shaped nanorod structures.** (**a**) The nETR spectra for V-shaped structures shown in Figure 3a with different gap widths compared with the single nanorod structure. (**b**) The nETR spectra for V-shaped structures with different corner parts for *g* = 10 nm and $r_{0}=20\sqrt{3}\;\mathrm{nm}$. (**c**) The nETR spectra for V-shaped structures shown in Figure 3b with different radii of the cylinder corner part and $r_{0}=20\sqrt{3}\;\mathrm{nm}$. (**d**) The nETR spectra for V-shaped structures shown in Figure 3b with $r_{0}=20\sqrt{3}\;\mathrm{nm}$ when the cylinder corner part is made of different materials; the case with *n* = 1 corresponds to the case with no corner part shown in Figure 3c. The other parameters are *θ*_1_ = *θ*_*D*_ = 60°, *θ*_2_ = *θ*_*A*_ = 60°, *L′* = 290 nm, and *d* = 20 nm.

In practical devices, it is very difficult to satisfy the parallel condition between the dipole moments of the donor and acceptor. In order to improve the RET rate for donor-acceptor pairs with nonparallel dipole moments, according to the above results, we propose new V-shaped structures. Figure 3a is the schematic picture of a V-shaped structure; the angle between the principle axis of each nanorod branch and the connection line of the dipoles are denoted as *θ*_1_ and *θ*_2_, respectively. For the dipole directions *θ*_*D*_ = 60° and *θ*_*A*_ = 60°, we also choose *θ*_1_ = 60° and *θ*_2_ = 60°, so the principle axis of each nanorod branch in this structure is aligned to a dipole. The distance from each dipole to the end of the nanorod is *d* = 20 nm. The height and width of each nanorod are set to be *a* = 40 nm. In order to improve the coupling between these two nanorods, we introduce a sharp corner part with gap widths *g* from the other ends of the nanorods. Figure 4a displays the nETR spectra for V-shaped structures shown in Figure 3a with different gap widths *g*, for *L′* = 290 nm, compared with the case of single nanorod. It can be seen that the nETR spectrum can be modulated by controlling the gap widths *g*. When the gap widths decrease, the resonance wavelength is red shifted, and the maximum enhancement increases. When *g* = 0 nm, the structure becomes whole, and the main resonance wavelength is remarkably red shifted and exceeds 1,800 nm. We can thus design the V-shaped structure with proper gap widths to obtain a nETR spectrum with approximately the same resonance wavelength as that in the single nanorod. Here we find that for the V-shaped structure with gap widths *g* = 10 nm, the nETR spectrum has a peak at 1,216 nm with an enhancement of about 80,200, which is about ten times that in the single nanorod. This indicates that, compared with the single nanorod, the V-shaped structure has a much stronger ability to enhance the efficiency of the RET between nonparallel donor-acceptor pair.

We then consider the structure with gap widths *g* = 10 nm for further optimization. To this end, we study a similar structure with different corner parts. The schematic picture of the structure with a cylinder-shaped corner part is shown in Figure 3b. The gap between each nanorod and the corner part was kept at *g* = 10 nm; the radius of the cylinder corner part is thus $r_{0}=20\sqrt{3}\;\mathrm{nm}$. The nETR spectra for these two V-shaped structures are displayed in Figure 4b. Compared with the structure with a sharp corner part, the nETR spectrum for the structure with a cylinder corner part has a lower maximum enhancement of about 76,200, while the resonance wavelength is almost unchanged. This indicates that as the gap widths are unchanged, the choice of the corner part shape has no important influence on the RET-enhancing ability of the V-shaped structures, which means that these structures have good fault tolerance in manufactory.

Even though the enhancing ability of the V-shaped structures is not influenced crucially by the shape of the corner part, the condition *g* = 10 nm here still requires the sophisticated control of the fabrication technology. In order to further reduce the difficulties in the fabrication process, we choose the V-shaped structure with a cylinder-shaped corner part shown in Figure 3b and consider reducing the radius of the corner so that the gap widths can be larger. The nETR spectra for different radii with $r_{0}=20\sqrt{3}\;\mathrm{nm}$ are displayed in Figure 4c, in which the center of the cylinder is unchanged. Compared with the case of radius *r*_0_, it can be seen that for the case of radius *r*_0_/2, the peak wavelength of the spectrum is blueshifted to 1,182 nm, and the maximum enhancement increases to about 82,100, while if the radius is further reduced to *r*_0_/4, the nETR spectrum does not show evident change any more. This indicates that when the radius of the cylinder corner part is less than *r*_0_/2, its influence to the nETR spectrum may be negligible.

On the other hand, we may also change the material properties of the cylinder corner part. The nETR spectra for different materials of the cylinder corner part are displayed in Figure 4d. Here the radius is set to $r_{0}=20\sqrt{3}\;\mathrm{nm}$ corresponding to the gap widths of *g* = 10 nm. The cases of material refraction index *n* = 1.5 and *n* = 3.4 are displayed together with the case of silver cylinder. We can see that when the material of the cylinder corner is changed, the resonance wavelength and the maximum enhancement in the nETR spectra both vary slightly.

The above results imply that the role of the corner part of V-shaped structures in nETR is minor. Based on this, we may remove the corner part so that the V-shaped structure consists of two nanorod branches only, as shown in Figure 3c. The nETR spectrum in this structure is also displayed in Figure 4d with *n* = 1; we can see that the resonance wavelength is 1,177 nm with a maximum enhancement of nearly 84,000. This resonance wavelength is very close to that in the case of single nanorod structure, while the maximum enhancement is ten times higher than the latter. Compared with other V-shaped structures having corner parts, this simple structure is thus more suitable to be applied in practical experiment and applications in integrated photonic devices.

**Figure 5:**
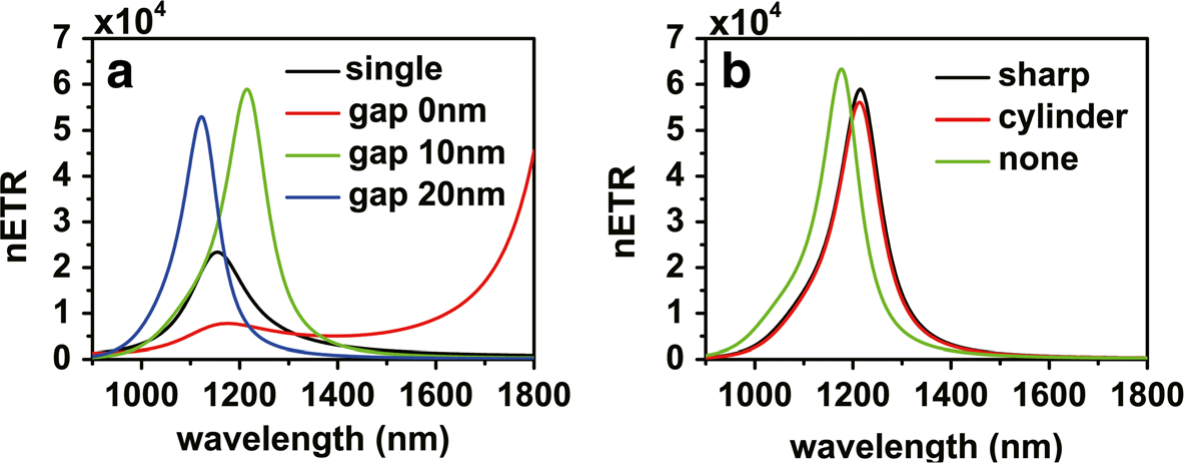
**The nETR spectra for different V-shaped nanorods structures, with*θ***_**1**_**=*θ***_**2**_**= 60°,*θ***_***D***_**= 60°, and*θ***_***A***_**= 30°.** (**a**) The nETR spectra for V-shaped structures shown in Figure 3a with different gap widths compared with the single nanorod structure. (**b**) The nETR spectra for V-shaped structures with a sharp corner part (black), cylinder corner part (red), or no corner part (green), *g* = 10 nm, and $r_{0}=20\sqrt{3}\;\mathrm{nm}$. The other parameters are *L′* = 290 nm and *d* = 20 nm.

In the above discussions, we proposed V-shaped structures with symmetric configuration for donor-dipole pair with symmetric dipole directions; the directions of the donor and acceptor dipoles are both aligned to the principle axis of the nanorod branches. In order to further examine the controllability and robustness of these V-shaped structures, we now discuss the RET-enhancing abilities of these structures for donor-dipole pair with asymmetric configuration *θ*_*D*_ = 60° and *θ*_*A*_ = 30°. Figure 5a displays the nETR spectra in the V-shaped structures shown in Figure 3a with a sharp corner part, *θ*_1_ = *θ*_2_ = 60°, and different gap widths *g*, compared with the case of single nanorod. Here we have *θ*_*A*_ ≠ *θ*_*D*_ and *θ*_*A*_ ≠ *θ*_2_; the direction of the acceptor dipole is thus a bit misaligned from the principle axis of the second nanorod branch. Compared with Figure 4a, the nETR in the single nanorod structure increases with a maximum enhancement of 23,300, while the RET-enhancing abilities of the V-shaped structures become weaker. Nevertheless, the nETR spectrum in the V-shaped structures can still be modulated by the lengths of the nanorod branches. The nETR spectrum in the V-shaped structure with a sharp corner part and *g* = 10 nm still has a maximum enhancement of about 59,000, stronger than that in the single nanorod structure. Figure 5b displays the nETR spectra for V-shaped structures with different corner parts shown in Figure 3 for *g* = 10 nm and $r_{0}=20\sqrt{3}\;\mathrm{nm}$. It can be seen that the RET-enhancing ability of the V-shaped structures is still robust. The nETR spectrum in the V-shaped structure consisting of two nanorod branches and no corner part has a maximum enhancement of 63,300 at wavelength 1,177 nm; this maximum enhancement is still nearly three times that in the single nanorod structures. The above results further demonstrate that the controllability and robustness of these V-shaped structures are preserved for donor-acceptor pair with asymmetric configuration.

## Conclusions

In summary, we have investigated the enhancement of the RET rate between donor and acceptor associated by the surface plasmons of the Ag nanorods on a SiO_2_ substrate. For donor-acceptor pair with parallel dipole moment directions, we have considered single nanorod with different cross sections, and the results revealed that the cylinder nanorod has the strongest ability to enhance the RET rate. We also found that the enhancement of RET rate in the single nanorod structure decreases for donor-acceptor pairs with nonparallel dipole moment directions. We then proposed simple V-shaped nanorod structures for nonparallel donor-acceptor pair. We demonstrate that the enhancement effect in these structures can be controlled by the nanorod length of the branch in the V-shaped structure. Our initial design of the V-shaped structure contains a corner part to improve the coupling between two nanorod branches, while we then find that the enhancement ability of the V-shaped structures is robust regardless of the shape and material of the corner part. Therefore, we may remove the corner part, and the V-shaped structure with two nanorod branches can lead to the remarkable RET rate enhancement that is ten times larger than that by the single nanorod. We also demonstrate that the controllability and robustness of these V-shaped structures are preserved for donor-acceptor pair with asymmetric configuration. Our work provides guidance on the application of simple nanorod structures to improve RET efficiency in integrated photonic devices.

## Abbreviations

nETR: Normalized energy transfer rate; RET: Resonance energy transfer.

## Competing interests

The authors declare that they have no competing interests.

## Authors’ contributions

YCY was responsible for the theoretical derivation, anticipated the numerical simulations, analyzed the simulation results, proposed the interpretation, and drafted the manuscript. JML performed the numerical simulations. CJJ and XHW conceived of the study and revised the manuscript substantially. All authors read and approved the final manuscript.

## Authors’ information

YCY and JML are PhD students at Sun Yat-sen University. CJJ and XHW are professors of Sun Yat-sen University.
